# An Overall Water Quality Index (WQI) for a Man-Made Aquatic Reservoir in Mexico

**DOI:** 10.3390/ijerph9051687

**Published:** 2012-05-04

**Authors:** Hector Rubio-Arias, Manuel Contreras-Caraveo, Rey Manuel Quintana, Ruben Alfonso Saucedo-Teran, Adan Pinales-Munguia

**Affiliations:** 1 Autonomous University of Chihuahua, Periferico Francisco R. Almada, Km. 1, Colonia Zootecnia, Chihuahua, Chihuahua, CP 31000, Mexico; Email: mcontrer2010@hotmail.com (M.C.-C.); rquintan@uach.mx (R.M.Q.); apinales@uach.mx (A.P.-M.); 2 National Research Institute for Forestry, Agriculture and Animal Production, Chihuahua, Chihuahua, CP 31890, Mexico; Email: saucedo.ruben@inifap.gob.mx

**Keywords:** WQI values, ANOVA, metals, water contamination, Chihuahua, Mexico

## Abstract

A Water Quality Index (WQI) is a useful statistical tool for simplifying, reporting and interpreting complex information obtained from any body of water. A simple number given by any WQI model explains the level of water contamination. The objective was to develop a WQI for the water of the Luis L. Leon dam located in the state of Chihuahua, Mexico. Monthly water samples were obtained in 2009; January 10, February 12, March 8, May 20, June 10, July 9, August 12, September 10, October 11, November 15 and December 13. Ten sampling sites were randomly selected after dividing the study area using a geographic package. In each site, two samples at the top depth of 0.20 m and 1.0 m were obtained to quantify physical-chemical parameters. The following 11 parameters were considered to calculate the WQI; pH, Electrical Conductivity (EC), Dissolved Oxygen (DO), color, turbidity, ammonia nitrogen, fluorides, chlorides, sulfates, Total Solids (TS) and phosphorous (P). The data analysis involved two steps; a single analysis for each parameter and the WQI calculation. The resulted WQI value classified the water quality according to the following ranges: <2.3 poor water; from 2.3 to 2.8 good water; and >2.8 excellent water. The results showed that the WQI values changed from low levels (WQI < 2.3) in some points during autumn time to high levels (WQI > 2.8) most of the year and the variation was due to time of sampling generally rainy season.

## 1. Introduction

An index is a single number that represents a large amount of data. For instance, the Water Quality Index (WQI) is a single numeric expression that interprets complex information obtained from any body of water, mostly related to water quality. Horton [1] at the middle of the past century, was the first researcher to suggest the advantages of calculating a WQI and since then, many studies concerning water indexes have been reported elsewhere for lake environments [2,3,4,5], river flows [6,7] and coastal areas [8,9]. These values are important when considering water use by humans and assessments for users or stakeholders. A WQI representing any water ecosystem can be affected by physical, chemical and biological factors [10,11].

In Mexico’s case, the availability of water shows a spatial and temporal variability. In southern Mexico, the state of Tabasco retains approximately 28% of the national water inventory [12]. By contrast, in northern Mexico, water is scarce, with some areas accumulating annual precipitation of less than 200 mm per year. Consequently, the water in these arid or semiarid environments is in short supply. The state of Chihuahua in northern Mexico has about ten million hectares with arid or semiarid environments [13]. In this particular state, water is the most critical natural resource and it has been hypothesized that the drought will worsen for two main reasons. The first being the high demand of more water by different sectors such as domestic, industrial, livestock, agriculture and others. The second reason is the high pressure the United States puts on water in accordance with the treaty between both nations [14]. The water problem is maximized when droughts occur like the one in 2011 in northern Mexico, which was considered the worst drought of the last 70 years. In addition, in some cases, a few rivers have been converted into dump sites with the potential consequence of negatively effecting human health and whole environments.

The Luis L. Leon dam in Chihuahua, Mexico is the downstream water reservoir that captures the water from the Conchos River. Different studies have been conducted in the Conchos River with the purpose of identifying its water quality in terms of physical-chemical-biologic parameters [15,16,17,18,19]. Moreover, metal and metalloid levels have been reported in the water as well as sediments throughout this river [20,21,22,23,24]. Nevertheless, the studies concerning the water in the Luis L. Leon dam are scarce or do not exist. The objective of this study was to develop a WQI for the water in the Luis L. Leon dam located in the state of Chihuahua, Mexico. The hypothesis was that water of this reservoir presents different levels of pollution throughout the year. These results are of vital importance to local inhabitants who will have a general knowledge of the water quality in their region during a certain period of time, instead of trying to understand complex water quality data. In addition, using this index will provide pertinent information to decision makers as to whether it is a benchmark-success or failure.

## 2. Materials and Methods

**Figure 1:**
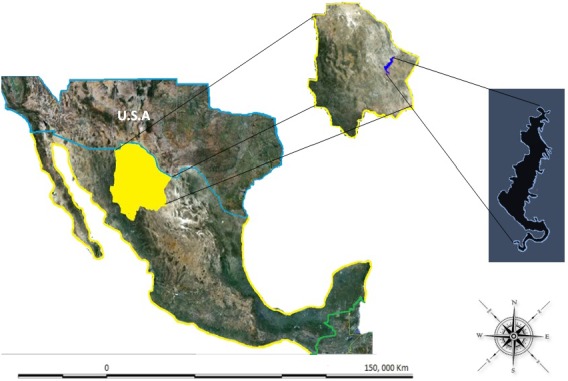
Location of the Luis L. Leon Dam in Chihuahua, Mexico.

The study was conducted during 2009 in the Luis L. Leon dam located in the municipality of Aldama, in the state of Chihuahua, Mexico. This dam was constructed between 1965–1968 with the aim of controlling water flow to irrigate the downstream municipality of Ojinaga, Chihuahua, Mexico [12] and to supply water to the United States according to the international treaty signed between the two countries [14]. This water reservoir is located at 105°19' and 105°24' North Latitude and at 28°52' and 29°00' East Longitude. The dam is located about 80 km from the capital Chihuahua and about 70 km from the city of Ojinaga that is on Mexican´s border with the United States of America (Figure 1). The total capacity of the dam is approximately 850 mm^3^ and the physiographic region belongs to the lacustrine area of the Sierra Madre Oriental.

During 2009, monthly water samples were collected on the following eleven dates; January 10, February 12, March 8, May 20, June 10, July 9, August 12, September 10, October 11, November 15 and December 13. The samples were collected at 10 random points of the dam area at the following depths; at the top (0–0.25 m) and at 1.0 m. The points were selected randomly after dividing the dam´s entire area into quadrants using a geographic package. At each point of the dam, two samples were obtained at two depths; 0.30 m and 1.0 m. Therefore, a total of 220 water samples were analyzed to quantify physical-chemical-metals.

The water samples were collected in polyethylene bottles according to Mexican normative [25,26,27]. A total of 38 parameters were detected in this study; yet only the following 11 parameters were considered in calculating the WQI; pH, Electrical Conductivity (EC), Dissolved Oxygen (DO), color, turbidity, ammonia nitrogen, fluorides, chlorides, sulfates, Total Solids (TS) and total phosphorous (P). Once collected, the water samples were transported directly to the laboratory at the College of Zoo-Technology and Ecology of the Autonomous University of Chihuahua and refrigerated at 4 °C for further analysis.

The parameters pH, EC and DO were measured *in situ* using portable measuring devices. The color was detected visually with the help of the Orbeco-Hellige model water tester, according to Mexican norm [28]. Turbidity was quantified according to the nephlometric method [29] using the Hanna HI 93703 turbid meter with a range of 0 to 1,000 Nephlometric Turbidity Units (NTU). Ammonia-N was measured using the macro-kjeldahl method [30]. Fluorides were quantified using the zirconil-SPADNS acid method, the chlorides were determined through the argent metric method [29] and sulfates were detected using the turbid metric method [29]. The TS parameter was detected using a Stabil-Therm soil test according to the Mexican norm [31]. The *p* value was detected using the colorimetric method using a Thermo Spectronic Genesys 20 spectrophotometer with a weave longitude of 625 nanometers [29].

The data analysis involved two general steps. The first was to conduct a statistical analysis (ANOVA) for any one of the 11 single parameters that were considered for the WQI. This univariate analysis was performed considering a factorial arrangement of treatments; the factor A was the sampling time with 11 levels (11 months) while factor B was the depth with two levels (0.25 m and 1.0 m). The second step was the aim of obtaining the WQI calculation which was developed according to the following three steps. The first step involved the transformation of the original data into standardized form with the purpose of achieving all dispersion data uniformly (0.1). During the second step, each of the 11 parameters included in the WQI were assigned with a specific weight (Wi) within a range of 1–4 according to the impact of that parameter in water quality where 4 was considered most important and 1 the least important. For this particular study, the number 4 was assigned to parameters pH, CE, OD and color; number 3 to the parameter turbidity; number 2 to the parameters ammonia nitrogen, fluorides, sulfates and chlorides and number 1 to the parameters TS and P. The third and final step was to allocate a level for each single parameter according to the analysis previously performed (Pi). The best level of water quality was assigned a low number and the worst levels a higher number. For instance, number one was given to values close to zero for those variables where the optimum water quality is closed to zero. The WQI was calculated with the following equation:


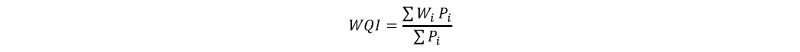


The calculated WQI could be classified according to the following ranges: <2.3 poor; from 2.3 to 2.8 good; and >2.8 excellent.

## 3. Results and Discussion

**Figure 2:**
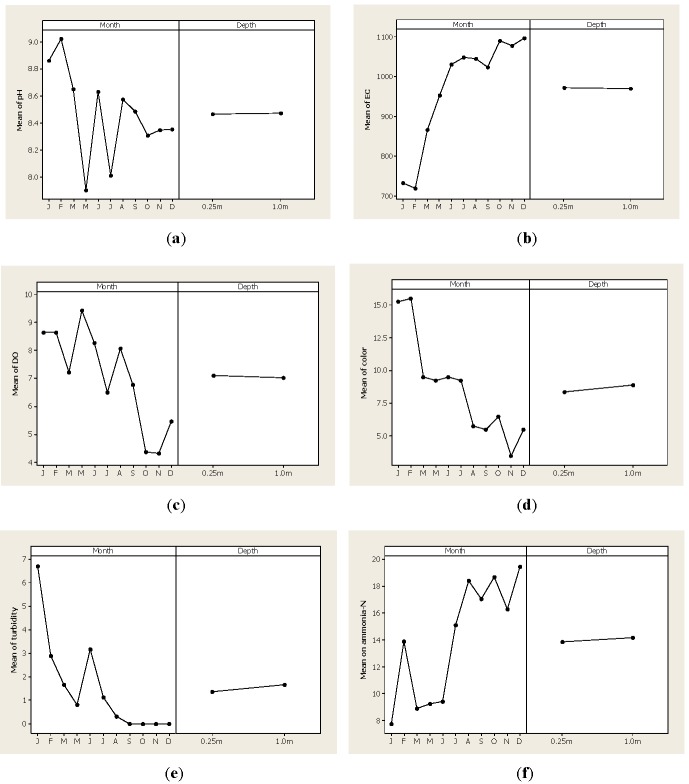
Level of the parameters pH, EC, OD, color, turbidity and ammonia-N detected in water samples of the Luis L. Leon dam in Chihuahua, Mexico.

**Figure 3:**
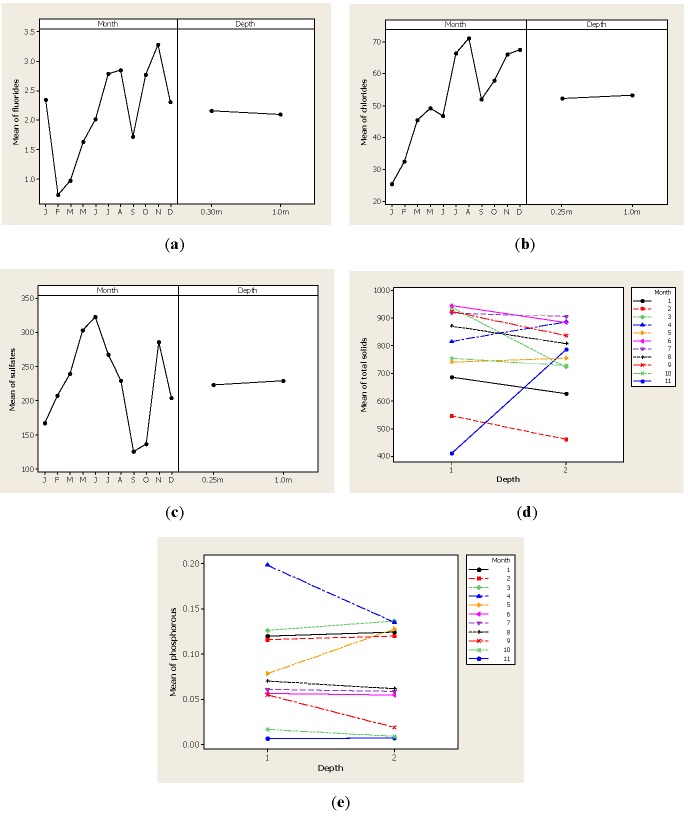
Parameter levels for fluorides, chlorides, sulfates, TS and P in water samples from the Luis L. Leon dam in Chihuahua, Mexico.

In general, the ANOVA detected differences for month sampling (*p* < 0.01) in nine of the variables tested for constructing the WQI. For the variables of total solids and phosphorous, there were differences in month sampling and also the interaction was significant (*p* < 0.01); however, the depth factor was not statistically significant in any variable (*p* > 0.01). Figure 2 gives out the results for pH, EC, OD, color, turbidity and ammonia-N as single parameters, while Figure 3 shows the results of the analysis for fluorides, chlorides, sulfates, total solids and phosphorous.

Figure 2(a) demonstrates that pH values ranged from 9.02 in February to 7.90 in May. These results are consistent with other studies performed at the Conchos’s watershed or the Conchos River [19,23,24]. Most of the time, the pH level was kept within the acceptable limits for potable or agricultural water but during January and February, the pH levels were above 8.86 which indicated the access of salts in the dam’s water. Levels above 9.0 might limit the physiology of some aquatic organisms and may affect the toxicity level of some heavy metals [32]. Figure 2(b) shows the EC results, in which lower levels were found in January and February, with 732.50 µScm^−2^ and 720 µScm^−2^ respectively. Yet, after the June samples, the EC values were higher than 1,000 µScm^−2^ in all months suggesting that there is some inorganic pollution. In other words, the EC levels increased after the rain period and this parameter is a good indicator of water quality [33] that is frequently included in WQI calculations [34,35]. It must be noted that higher EC levels do not mean that the water will present a health issue but it can be indicative of the amount of dissolved chemicals in the water.

Figure 2(c) shows the DO levels detected in the Luis L. Leon dam water where higher values were quantified in May with 9.43 mg·L^−1^ and lower values were observed in October with 4.32 mg·L^−1^ and November with 4.32 mg·L^−1^. In general, the OD is kept at a good level during the entire year (>5.0 mg·L^−1^); nevertheless, during the autumn samples, the OD is low but not dangerous to aquatic life (<3.0 mg·L^−1^). It is generally accepted that DO is consumed by oxidation in organic matter in water and a low OD level might increase the toxicity of some heavy metals and some pesticides [36]. The lowest concentration of DO level after the rainy season indicates a contamination of the dam water by runoff from agricultural lands established at the highest parts of the watershed. This is because after precipitation events the water is rich in organic matter and other elements and therefore, bacteria uses oxygen to biodegrade it.

Figure 2(d) shows the color levels detected in water samples, where lower values were detected during the November samples with 3.5 CU while higher values were detected in the January (15.25 CU) and February samples (15.50 CU). Figure 2(e) shows that turbidity was higher at the beginning of the year with 6.70 NTU and then it was lowered to reach levels of zero during the latter part of the year. The turbidity comes from clay particles within the eroded soil in any catchment area and is routinely utilized to indicate drinking water quality. Other factors such as microbiological contamination are correlated with water turbidity. Limits of acceptable turbidity for drinking water vary between countries but in general are below 2 NTU. For this reason, outbreaks of gastrointestinal illness have been associated with high turbidity levels [37,38].

Figure 2(f) shows the concentration of ammonia-N levels during the year with lower values of 7.76 mg·L^−1^ noted in the January samples while the latter part of the year presented higher values with a concentration of 19.42 mg·L^−1^ detected in December. These concentrations are above the maximum levels established in the Mexican normatively for potable water (0.05 mg·L^−1^) as well as for agricultural water (0.06 mg·L^−1^). Higher levels of ammonia-N were observed after the August samples that coincided with the rainy season and these findings were important because levels above 5 mg·L^−1^ might be toxic to some aquatic life. Figure 3(a) shows the concentration of fluorides where higher concentrations were detected in the November samples with 3.28 mg·L^−1^ while lower concentrations were observed in the February samples with 0.72 mg·L^−1^ and March samples with a level 0.97 mg·L^−1^. Our findings concerning fluoride concentration are similar to those reported by Sabahi *et al*. [39] who worked with surface water in Yemen and found levels in a 3.30 to 3.72 mg·L^−1^ range. This trend was also noted the concentration of chlorides (Figure 3(b)) detected in the water samples of the Luis L. Leon dam where the highest concentration was detected in August with 71.23 mg·L^−1^. Figure 3(c) shows the presence of sulfates, with the highest peak in the May and June samples while the lowest values were in the September and October samples. As previously explained, the analysis for TS and P parameters detected a significant interaction between month and depth factors that is shown in Figure 3(d) for TS and in Figure 3(e) for P. As expected, the highest concentration of TS was measured after the rainy season while P levels were higher in the March and May samples.

**Figure 4:**
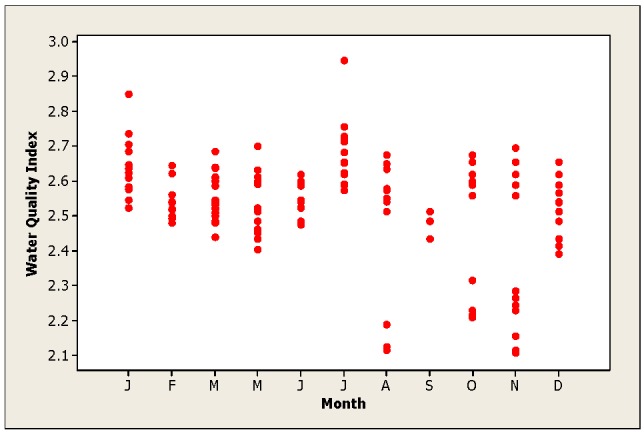
WQI values calculated during different months in the Luis L. Dam in Chihuahua, Mexico.

**Figure 5:**
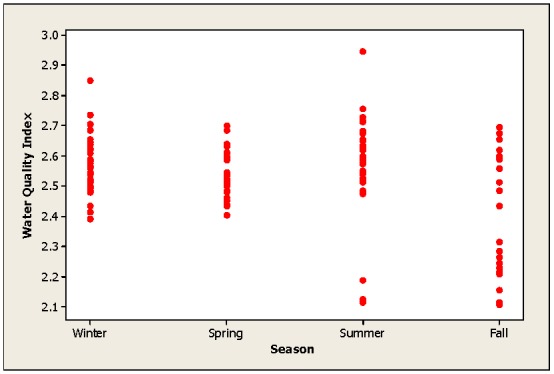
WQI values calculated for the fourth season in the Luis L. Dam in Chihuahua, Mexico.

Figure 4 displays the WQI detected in the Luis L. Leon dam in Chihuahua, Mexico. Generally, the water could be categorized as good during the entire year. Nonetheless, the August and November samples displayed some points that could be categorized as poor. To better understand the WQI, Figure 5 depicts the index calculated during the fourth season of the year. During summer and autumn, some water points could be classified as poor. We have to assume that dynamic urban growth, increased industrial actions, intensive farming and milk production industries as well as high fertilizer applications in agricultural production above the Luis L. Leon Dam are responsible for the changes in the water quality. In essence, the changes in water quality are due to these anthropogenic effects. De la Mora *et al*. [5] developed a WQI for Chapala Lake in Jalisco, Mexico and concluded that the water quality is altered mostly by rain events.

Alobaidy *et al*. [2] designed a WQI for the Dokan Lake in Iraq and found that water was categorized as good water for four years; but the 2009 water sampling was considered poor. In other study, Yisa and Jimoh [6] calculated a WQI for the water of the Landzu River in Bida, Nigeria and concluded that there was no acceptable drinking water in any sample taken because the water quality was not within the permissible limits. These authors categorized water quality into five types from excellent to unsuitable for drinking. Rejith *et al*. [40] designed a WQI for groundwater for drinking purposes in India. They used the following three classifications; poor (<10), moderate (10–20) and good (>20) and found that most water could be categorized from moderate to good. Rabee *et al*. [41] found that the water of the Tigris River was moderate throughout the season. These authors detected a high value WQI during the spring while the lowest value was recorded during autumn.

## Conclusions

During the last decade the Conchos watershed has been subjected to rapid decline in water quality, and this trend is understandable due to the increases in the human population and activities throughout the watershed. This study confirms this statement, which showed that most parameters increased after the rainy season. Moreover, the calculated WQI reiterates this effect that water quality declined after the rainy season. These results are important so that the local authorities may implement preventive measures to reduce the threat of domestic and industrial discharges as well as agricultural activities’ discharges.
